# Cancer patients’ satisfaction with telehealth during the COVID-19 pandemic

**DOI:** 10.1371/journal.pone.0268913

**Published:** 2022-06-03

**Authors:** Samantha R. Paige, Gemme Campbell-Salome, Jordan Alpert, Merry Jennifer Markham, Martina Murphy, Eve Heffron, Chris Harle, Sijia Yue, Wei Xue, Carma L. Bylund

**Affiliations:** 1 Dept. of Health Outcomes and Biomedical Informatics, University of Florida, Gainesville, Florida, United States of America; 2 Geisinger Medical Center, Danville, Pennsylvania, United States of America; 3 College of Journalism and Communications, University of Florida, Gainesville, Florida, United States of America; 4 Division of Hematology and Oncology, University of Florida, Gainesville, Florida, United States of America; 5 Dept. of Biostatistics, University of Florida, Gainesville, Florida, United States of America; All India Institute of Medical Sciences - Bhopal, INDIA

## Abstract

**Objective:**

To examine factors associated with cancer patients’ satisfaction using telehealth during COVID-19, including video conferencing platforms and secure messaging systems.

**Method:**

Patients with cancer participated in a cross-sectional, web-based survey was conducted with patients with cancer. The survey included questions about satisfaction with video-conferencing and secure messaging platforms to interact with clinicians during the COVID-19 pandemic. Logistic regression analyses were conducted to examine predictors of satisfaction for each telehealth platform.

**Results:**

Participants generally reported positive satisfaction with each telehealth platform. Both platforms were commonly used to review medical results and discuss symptoms or treatment. Participants identifying as a man were most satisfied with their video-conferencing session, especially if they had a comfortable place to sit. Patients were more satisfied with secure messaging because they could ask a question without scheduling an appointment.

**Discussion:**

When strategically used together, video-conferencing platforms and secure messaging may increase patient satisfaction in cancer care during the remainder of the pandemic and beyond. Attention must be paid to optimizing factors that promote satisfaction for each telehealth platform.

## Introduction

Risk mitigation strategies (e.g., physical distancing, mouth coverings) have been widely adopted in response to COVID-19. People who are immunosuppressed (e.g., undergoing cancer treatment) are at an especially high risk for the negative outcomes associated with COVID-19 (e.g., hospitalizations, 30-day mortality) [1]. As a result, telehealth has become a primary healthcare delivery solution in oncology settings [2]. Telemedicine, for example, allows patients and clinicians to interact at a distance via HIPAA-compliant video-based platforms and secure message systems [3]. Patients are provided a web-link (or URL) to access at their predetermined appointment time. Once they access the platform, patients are instructed to wait until their clinician joins the call which is when the appointment will begin. Oncologists have acknowledged both the benefits (e.g., convenience) and drawbacks (e.g., ability to build effective alliances with patients) of delivering care via telemedicine [2,4]. Whereas patients with cancer have generally reported positive aspects of telemedicine, including its convenience and perception that the technology has improved their health in some capacity [5,6].

Patient satisfaction is a proxy measure for healthcare quality that gauges the perceived effectiveness of care and experience interacting with clinicians [7,8]. Shaverdian and colleagues [9] conducted a study with radiation oncology patients and found no statistically significant difference in patient satisfaction between telemedicine and in-person office visits during the pandemic. In fact, most patients preferred telemedicine over in-person care (45% vs. 34%) and 21% did not find any difference in the two settings. Healthcare systems use patient experience (i.e., satisfaction) measures to identify areas of exemplar service and areas of service that require improvement [9]. Although researchers have demonstrated a positive association between telemedicine usability and patient satisfaction [6], little is known about the patient-oriented factors that predict patient satisfaction with telehealth in oncology settings during the pandemic. Such factors include the type of technological device used to communicate with oncology clinicians, as well as the purpose of the appointment and general comfort using the technology to interact with their clinician. The purpose of this study was therefore to examine factors associated with cancer patients’ satisfaction using telehealth during COVID-19, including video conferencing platforms and secure messaging systems.

## Methods

### Sample and procedures

Eligible participants were individuals 18 years old or older who had been diagnosed with cancer and had at least one video conference visit with a clinician between March 2020 and the time of data collection (July 29-Sept. 2, 2020). We recruited participants to participate in a web-based survey hosted by REDCap^®^. We used three recruitment methods: (1) a broad consent research registry consisting of patients who volunteered to be contacted about local research opportunities for which they are eligible; (2) an online posting on The Leukemia & Lymphoma Society’s (LLS) online community; and (3) an e-mail recruitment distribution to members of the International Waldenstrom’s Macroglobulinemia Foundation (IWMF) by the organization. Individuals who completed the survey were offered a $25 eGift card for their time. The University of Florida’s Institutional Review Board (IRB) approved this study (IRB#202001641). Participants completed an online informed consent prior to data collection, and all data were collected, de-identified, and analyzed anonymously.

### Measures

Participants reported their age, gender, race, ethnicity, and highest level of education. They also identified their cancer(s) and the length of time they have lived with a cancer since completing the survey. Participants answered questions about their experience using telemedicine. They answered whether they have used a video consultation or secure messaging to communicate with their clinician since March 2020, and they identified the device they had used for the consultation (e.g., computer, tablet, smartphone, other). For video conference consultations, participants reported the number of times they have used the technology, what specialist they had seen remotely, and whether they had previously seen the clinician in-person. They also answered four questions about whether the video conference technology was easy to use, if they were able to overcome technical difficulties with their clinician, if they had a comfortable place to sit during the consultation, and whether the camera was well-placed to easily see the clinician’s face (1 = strongly disagree to 5 = strongly agree). Finally, participants answered two questions about their satisfaction using the video conference and secure messaging platforms (1 = very dissatisfied to 5 = very satisfied). Responses to satisfaction items were left-skewed and were ultimately dichotomized (0 = very dissatisfied, dissatisfied, neither satisfied or dissatisfied; 1 = very satisfied, satisfied).

### Data analysis

Datasets from both samples were combined after conducting chi-squared tests that resulted in statistically non-significant differences in key variables. Frequency statistics were conducted to describe the sample. We conducted a lasso regression to model how telemedicine satisfaction was associated with demographics, technological device used, cancer diagnosis, and telemedicine appointment purpose. Then, we conducted a logistic regression with the variables selected from lasso regression. The second lasso regression model was conducted to model how demographics, cancer diagnosis date and security message purpose associate with patient satisfaction using secure messaging systems. We included variables that did not shrink to 0 from the lasso regression in the logistic regression models. All tests were two-sided and p-values less than 0.05 were deemed statistically significant. R version 4.0.2 was utilized for all the data analysis work.

## Results

Table 1 shows the socio-demographics of the sample. Participants were adults 19 to 88 years old (*M* = 64.21; *SD* = 12.98) and 49.4% identified as female. More than three-quarters (78.2%) of participants were White and 5.3% were Black/African American. Only 2.9% identified as Hispanic. Nearly half of participants (43%) completed some college or earned an associate’s or bachelor’s degree, 35.9% completed some postgraduate coursework or earned at least a master’s degree, and 7.1% earned a high school/vocational degree.

**Table 1:** Demographic characteristics of patients with cancer.

Demographic	*n* (%)
**Sex**	
Female	84 (53.16)
Male	61 (39.87)
Do not wish to answer	1 (0.63)
Missing	12 (7.59)
**Race**	
White	133 (84.18)
African American	9 (5.70)
Other	3 (1.90)
Missing	13 (8.23)
**Education**	
High school	9 (5.70)
Other degree	13 (8.23)
Bachelor	63 (39.87)
Advanced degree	61 (38.61)
Missing	12 (7.59)
**Marital Status**	
Married	109 (68.99)
Not married	14 (8.86)
Other	22 (13.92)
Missing	13 (20.89)

Nearly one-third (32.9%) of participants were diagnosed with Waldenstrom’s Macroglobulinemia, 11.8% with breast cancer, 3.5% with Non-Hodgkin’s Lymphoma, and 4.1% with multiple cancers. Participants reported *M* = 2497.40 (*SD* = 3876.93) days since a diagnosis. Participants met with a member of their healthcare team remotely *Mdn* = 1 time (interquartile range [*IQR*] = 1) since March 2020. Nearly three-quarters (74.1%) of participants had seen their clinician in-person prior to their remote appointment *Mdn* = 10 times (*IQR* = 16). Most participants (85.9%) were enrolled in a patient portal where secure messages are exchanged with a clinician. Table 2 shows that participants met with their clinician via a computer (58.8%), smartphone (25.9%), or tablet (14.1%). Over half (64.7%) reported speaking with a medical oncologist during their video conference appointment, whereas 5.9% of participants spoke with an oncology surgeon and 6.5% with a radiation oncologist. Approximately 60% of participants used video-conferencing calls to review results with their clinicians, whereas 36% discussed symptoms and 24% discussed treatment. Among participants who used secure messaging to communicate with their clinician, the primary reasons were to review results (36%), decide a treatment (13.03%), or receive more information about a treatment (13.03%).

**Table 2:** Device, telemedicine type, and purpose of remote medical appointment.

Variable	*n* (%)
**Type of Device**	
Computer	100 (63.29)
Phone	34 (21.52)
Tablet	22 (13.92)
Other	2 (1.26)
**Clinician Seen via Telemedicine**	
Medical Oncology	110 (69.62)
Radiation Oncology	11 (6.96)
Surgery	10 (6.33)
Other	18 (11.39)
Not Sure	7 (4.43)
Missing	2 (1.27)
**Purpose of Video Appointment**	
Discuss Symptoms	57 (36.08)
Diagnosis	22 (13.92)
Review Result	91 (57.59)
Make Decision about Treatment	30 (18.99)
Treatment	38 (24.05)
Other	30 (18.99)
**Purpose of Secure Message** ^a^	
Discuss Symptoms	13 (8.90)
Discuss a Diagnosis	9 (6.16)
Review Result	53 (36.30)
Make Decision about Treatment	19 (13.03)
Receive Treatment	19 (13.03)
Ask a COVID-19 Question	16 (10.96)
Other	42 (28.76)

Note.

^a^Based on 146 participants who said they are enrolled in a secure messaging system.

On average, participants agreed or strongly agreed that the video conference technology was easy to use (*M* = 4.26; *SD* = .77), they were able to overcome technical difficulties with their clinician (*M* = 4.44; *SD* = .56), they had a comfortable place to sit (*M* = 4.46; *SD* = .59), and the camera was well-placed to easily see the clinician’s face (*M* = 4.03; *SD* = .84). Participants also reported they were satisfied with using a video conference platform (*M* = 4.46; *SD* = .90) and with using secure messaging (*M* = 4.34; *SD* = .89) for healthcare during the pandemic.

Table 3 shows the results of the logistic regression of the video conferencing platform satisfaction. Participants who agreed/strongly agreed that they had a comfortable place to sit during their video-conferencing appointment were 8.88 times (95% CI: 1.03–76.60) more likely to be satisfied with their video appointment. Compared to female participants, males were 5.12 times (95%CI: 1.06–24.74) more likely to be satisfied with video appointment. Although not statistically significant, participants whose purpose of the video conferencing session was to discuss symptoms related to diagnosis or treatment were 3.49 times (95% CI: 0.72–16.89) more likely to be satisfied than participants with another purpose.

**Table 3:** Predictors of video-conferencing appointment satisfaction.

Predictor	Odds Ratio	95% CI		P Value
Intercept	0.31	0.04	2.51	0.27
Have a comfortable place to sit^a^	8.88	1.03	76.60	0.05
Gender^b^	5.12	1.06	24.74	0.04
Device: Computer^c^	2.64	0.80	8.75	0.11
Purpose: Discuss symptoms^c^	3.49	0.72	16.89	0.12

Note.

^a^ Agree vs. Disagree;

^b^Male vs. Female;

^c^Yes vs. No.

Table 4 shows the results of the logistic regression of secure messaging system satisfaction. The days from diagnosis is calculated from the difference of the diagnosis date and the date of completing the survey. Since it is significantly left skewed and not normally distributed, the log transformation is included for this predictor. There was no statistically significant difference of the secure message satisfaction in age, gender or cancer diagnosis dates. Participants whose purpose of utilizing secure message was to ask a question that did not require an appointment were 3.31 times (95% CI: 1.00–10.95) more likely to be satisfied than participants who reported other purposes. In addition, participants who always use secure messaging to communicate with their healthcare team were 11.84 times (95% CI: 1.32–106.16) more likely to be satisfied than others.

**Table 4:** Predictors of secure message satisfaction.

Predictor	Odds Ratio	95% CI		P Value
Intercept	0.92	0.02	51.97	0.97
log(days from diagnosis)	0.86	0.53	1.38	0.53
Gender^a^	0.72	0.23	2.31	0.58
Age	1.04	1.00	1.08	0.05
Purpose: Question that didn’t need appointment^b^	3.31	1.00	10.95	0.05
Purpose: always use secure messaging to communicate with my oncology team^b^	11.84	1.32	106.16	0.03

Note.

^a^Male vs. Female;

^b^Yes vs. No.

## Discussion

Patients living with cancer reported a high degree of satisfaction interacting with their clinicians via telehealth platforms, including video-conferencing and secure messaging platforms. Patients generally used a desktop computer to communicate with their clinicians. Video-conferencing platforms were used to review results and discuss symptoms, whereas secure messaging platforms were used to review results and learn more about treatment. Satisfaction with either video-conferencing or secure messaging platforms was dependent on different factors, demonstrating the need to explore a variety of strategies to optimize patient satisfaction with each platform.

Patients with cancer reported high satisfaction using video-conferencing platforms to communicate with their clinicians. Patient satisfaction increased when patients reported that they discussed symptoms via video-conferencing platforms with their clinicians. Discussing symptoms can be an emotional experience and sharing this information without immediate, comforting feedback may exacerbate distress experienced by patients [10]. Further, something very simple as having a comfortable place to sit may make a big difference in patients’ satisfaction using video-conferencing platforms. This finding emphasizes the importance of clinicians maintaining face-to-face interactions via videoconferencing with patients, especially during times of crisis (i.e., a pandemic). Healthcare systems could suggest when they send out reminders and instructions about telemedicine visits to find a comfortable place to sit.

Patient satisfaction with secure messaging was strongly predicted by perceptions about the convenience of direct, asynchronous communication. During the pandemic, patients used secure messages to communicate with their clinicians about changes and adjustments related to scheduling care, as well as advice related to risk of COVID-19 and precautions for visiting healthcare systems [11]. Prior research has demonstrated that patients with cancer value secure messaging as an option to reach their clinicians and believe it may be equally impactful to their care as in-person interactions [12]. Satisfaction and utilization of secure messaging may facilitate patient-clinician communication between appointments, supporting patient well-being and adherence. This should be explored further.

Men reported greater satisfaction using video-conferencing platforms to receive cancer care. This finding is consistent with research demonstrating that women are less likely to use and report satisfaction using video-conferencing platforms for medical care [13]. Women have been more likely to delay or avoid medical attention during the COVID-19 pandemic [14]. Disparities in telemedicine utilization have been linked to increased informal caregiving (i.e., parent, child) duties and unemployment [14]; however, these factors do not explain why women are more likely to report poor satisfaction with the video experience. This will be an important area of research to support gender equity in telemedicine.

Regarding secure messaging, neither gender nor age were statistically significant predictors of satisfaction using secure messaging. Also surprising is that time since cancer diagnosis was not associated with satisfaction using this communication modality to communicate with oncology clinicians. A recent study found that patient portals are commonly used by patients with an existing cancer diagnosis (i.e., younger, white, and higher socioeconomic status) to review test results [15]. Efforts to further segment patients with cancer according to their point on the care continuum, beyond simply date of diagnosis, will be important to understand the nuances of cancer care and secure message use.

### Limitations

This was a cross-sectional, web-based survey at a specific time-point early in the COVID-19 pandemic. These results therefore cannot be generalized to the entire pandemic. Surveillance of patient satisfaction and common predictors should be maintained to detect any trends due to social policy changes or intervention. Future research should also consider patient satisfaction with telemedicine according to where they are in their patient journey, such as active treatment, palliative therapy, or completed active therapy with long-term follow-up. Data in this study were drawn from a local sample with heterogeneous cancers and one from a national sample with the same diagnosis. This provides generalizability to our findings, but also demonstrates the need for investigating telemedicine satisfaction and experiences among patients with specific cancers to support targeted efforts. Further, it will be important for future research to explore how patient perceptions about the confidentiality and privacy of telemedicine appointments and related out-of-pocket expenses influences their satisfaction using the modality.

### Conclusions

Patients with cancer report satisfaction using video conferencing platforms and secure messaging systems to communicate with their clinicians during the COVID-19 pandemic. Satisfaction with video-conferencing platforms was greatest among men and patients who used the platform to talk about symptoms with their oncologist. Patients were satisfied using secure messaging because it was a quick and convenient alternative to making an appointment with their clinician. When strategically used together, video-conferencing platforms and secure messaging may increase patient satisfaction and have a downstream effect on telehealth utilization and improved health outcomes.

## Supporting information

S1 Dataset (XLSX)
